# Our Imperfect Culture

**Published:** 1889-02

**Authors:** 


### Editorial.

### OUR IMPERFECT CULTURE.

It is a well-known fact that the Americans are essentially a practical people, a people who are accustomed to look at the utilitarian aspect of a question, rather than at its purely intellectual phases. Thus it happens that their inventions usually show the greatest ingenuity; and in the application of labor-saving machinery, which are marked by both speed and excellence, they lead the world. The same can be said even of the learned professions. The skill of American surgeons is known and appreciated the world over. This does not apply to surgery alone, but to the American dentist, also, for the excellence of American dental operations is proverbial. So much so, that many have used the title "American Dentist" with the view of increasing their patronage and thereby their incomes.

The medical course in Europe is longer than it is in this country, yet we find that the American graduate is not only fully equal in skill and the practical application of the principles of medicine, but that, in even competition is usually more successful in winning practise than his European rival.

We noticed a short time since an article from the pen of a distinguished professor in an Italian school of medicine, in which he remarked, that when he came over here to examine our schools he was at first prejudiced against them on account of their short courses, yet, that after a careful study of the methods of teaching prevalent here, and of the aptness of the students, he had come to the conclusion that the average American student could learn as much in three years, *caeteris paribus*, as the Italian could in four. But despite of the fact that the American mind and the American genius are not a whit less than those of any other country, yet it undoubtedly remains true, that the palm for accurate and exhaustive physiological and pathological research does not rest with us. And why not? We find the explanation partly in that peculiar spirit innate in an American, which urges him to do in haste that which he is able to perform. Everything is done apparently with the intention of economizing time; eating, drinking, travelling, all the work of both the artisan and professional man are done under high pressure; even our pleasures are taken with a rush. Such habits and surroundings are utterly inimical to the slow and plodding processes of methodical research. Where in all America could we find a man like one wellknown German, who spent a lifetime in studying the Latin cases, and then regretted on his death-bed that he had not confined his attention entirely to the Dative case; or, like another, pass his days in unravelling the intricacies of the second Aorist tense? Our nervous characteristics are mainly due to our surroundings, and to our rapid growth as a nation, so that we are much like the precocious child at school—having an ability ahead of our years, but not the physical qualities necessary to make proper use of that ability.

Although the reason for our deficient culture, to a certain extent, lies in what has just been said, the main reason we think, can be ascribed to a lack of the facilities and endowments which, the institutions in other countries equal in the scale of civilization and culture with ours, have. Other countries have their huge piles of buildings, universities, which are made comfortably secure from financial embarrassments either through government aid or private endowments, and about which are gathered galaxies of the most ambitious young men of that particular country. And should one of these men distinguish himself in his peculiar branch, he will receive an appointment which secures a living, and throughout the remainder of his life he may, if he choose, devote himself to studying the growth of a hair or the development of a tooth, secure from the trials that beset a man who must from day to day, earn his own livelihood. And is such a life of no benefit? It certainly is, for these are the men who are quoted the world over, who mould the opinion of the educated world, and whose work is most likely to go down the ages.

If a man can, at this advanced stage of civilization, give to the world one new idea, or make one useful discovery or invention, he has been of value not only to his fellowmen, but to countless succeeding generations, and his life has been well spent. Not all persons, however, care to pass their lives in the plodding quietness of scientific research; but there are some who do, and for these there ought to be provided suitable means.

Now, these accurate investigations are not remunerative from a pecuniary point of view, yet they are essential, and he who makes them must live. And, further, the apparatus and the materials necessary for making many of these investigations are so costly, that few private individuals are able to purchase them, unaided; and it is well known that the wealthiest men are not generally those who are willing to devote their lives to an occupation which entails so much hard work and gives promise of so little return. There are only two ways by which Americans can gain a prestige in accurate scientific investigation equal to that which they already possess in the practical application of the knowledge gained by such investigations in other countries, and those ways are either through a system of government bureaux created for special investigation or through private endowment.

For our own part we think that our government would do well to imitate the example of most of the other civilized countries, by creating positions for those who have shown by their work that they should have full time and means to pursue such inquiries as will redound to the benefit of mankind. But many think such a course at variance with the genius of our institutions; and, whether it is or not, we do not have a system of government pensions, and must therefore depend on the only other means left—that of private benefactions.

OUR INSTITUTIONS.

It is greatly to the credit of our wealthy men that we have many noble institutions of learning, the result of their generous gifts. Among these may be mentioned Harvard, Yale, Princeton, Johns Hopkins, the University of Pennsylvania, and many other institutions which are gathering about them the most distinguished men of letters and science. Johns Hopkins University is to be particularly commended, for it is the first institution of our country to adopt the experimental scientific methods of European institutions. From the good work done in the past, and from the assemblage of learned men now gathered within its walls, we may expect great advancement in the future. This is an instance of a school that is heavily endowed, and can afford to pay first-class salaries to first-class men.

The College of Physicians and Surgeons of New York received a fine gift from the Vanderbuilt estate, and with this they have erected the finest laboratories in the world. But this large sum will not last forever, and the College of Physicians and Surgeons even now needs the interest on another million to pay salaries to the men who should work in those laboratories. The present tendency is toward the better endowment of our institutions as witnessed by the recent founding of the Leland Stanford, Jr., University of California, which has received a gift of astonishing magnificence—aggregating 15 millions of dollars well invested—the income of which is to be devoted to the upbuilding of a University second to none other in the world. As the prospectus says "the brief history of California, as an American state, comprises much that is noble and great, but nothing in that history will compare in grandeur with this act of one of her leading citizens. The records of history may be searched in vain for a parallel to this gift of Senator Stanford's to the state of his adoption. The utter absence of ostentation and the singleness of purpose which have characterized this gift of many millions, render the act unique in the records of public benefactions. Many wealthy persons have in the evening of their days * * * * or by will after death bestowed large portions of their wealth for the public benefit; but in this case the donor is a man hardly past the prime of life, in robust health and in the full strength of unimpaired faculties * * * * yet he freely gives a large part of his more than princely wealth to advance the cause of education, and afford the sons and daughters of California ample opportunity for obtaining the highest and broadest culture."

This university can truly be called great; for surely the title will be deserved by a pile of buildings which are to be erected as a noble memorial to the only son and child of Senator Stanford, Leland Stanford, Junior, now deceased. Senator Stanford has devoted much time and thought to the elaboration of this project, having first secured an act of legislature with regard to endowments for educational institutions, and he intends that this school shall in time be equal to, and if possible excel, all the other best colleges of the world. We say, "in time," for Senator Stanford very wisely does not desire that the money shall at once be consumed in the erection of great piles of buildings, out of proportion to both the number and the wants of the students; but he intends that his undertaking shall be a normal growth. The act of endowment requires that within two years the trustees shall adopt a general plan for the university buildings, and that "such buildings shall be plain and substantial in character, and extensive enough to provide accommodations for the university and the colleges, schools, seminaries, mechanical instituties, laboratories, conservatories and galleries of art part thereof." It is further stipulated that the buildings shall be built only as fast as they are needed, and not faster, the Senator sensibly adding that extensive and costly buildings do not necessarily make a university; but that it depends for its success rather on the character and attainments of its faculty. With regard to the faculty, the founder intends that it shall be the best attainable, and to that end has provided that higher salaries shall be paid in this university than in any other similar institution in the world. Here are to be erected schools of medicine, dentistry, law, mechanics, indeed of every known branch of learning; and the result of these exceptional salaries, coupled with the other advantages afforded, will be to collect about each of these schools the most learned men of that particular branch. When we add to the inducement of salary the fact that California probably has the most equable and pleasant climate of the civilized world, and that here the student and the scientist will be separated from the hurly burly of the social and the business world, both so inimical to close application, we have all the factors that tend towards the solving of the more recondite mysteries, and the steadily pursuing of the more untrodden paths of science. So we earnestly hope that part of this great sum will be devoted to the erection of such laboratories, and with such apparatus, that the most eminent and studious men may there pass their lives in physiological and biological research, fields of extraordinary interest, and the results of which are of vital importance to every human being.

The trustees of the University are to concern themselves mainly with the financial affairs pertaining thereto, while the president of the school is to have the exceptional powers of "prescribing the duties of the professors and the teachers; of removing professors and teachers at will; and of prescribing a course of study and the manner of teaching;" Senator Stanford's idea being that the president should be responsible to the trustees for the educational management of the institution, and to make him absolute, with the other members of the faculty as his staff, believing that power and responsibility go together. A number of free scholarships are to be given either to those who merit them by their conduct and study, or to the deserving children of those who, dying without means in the service of the State or in the cause of humanity, have a special claim on the good will of mankind. Thus these scholarships will not be given as charities, but as benefits both deserved and earned. Post-graduate courses of lectures are to be delivered by the ablest professors on the various subjects of advanced learning, and these lectures are to be free not only to the graduates of the University, but also to the graduates of other schools. One of the stipulations, and a good one, is that the doors be open to both male and female alike, since the donor considers it of the first "importance that education be full and equal, and varied only as nature dictates."

In short, Senator Stanford intends that this shall be a fount of learning that shall satisfy the cravings for knowledge of the humblest mechanic to the most finished scholar or scientist. This great institution, if continued on as broad a base as its founder evidently contemplates, must in the future surely give forth much that is valuable to mankind, and we shall, with great interest, observe its progress.

